# A Review of Colorectal Cancer in Terms of Epidemiology, Risk Factors, Development, Symptoms and Diagnosis

**DOI:** 10.3390/cancers13092025

**Published:** 2021-04-22

**Authors:** Tomasz Sawicki, Monika Ruszkowska, Anna Danielewicz, Ewa Niedźwiedzka, Tomasz Arłukowicz, Katarzyna E. Przybyłowicz

**Affiliations:** 1 Department of Human Nutrition, Faculty of Food Sciences, University of Warmia and Mazury in Olsztyn, Słoneczna 45F, 10-719 Olsztyn, Poland; monika.ruszkowska@uwm.edu.pl (M.R.); anna.danielewicz@uwm.edu.pl (A.D.); ewa.niedzwiedzka@uwm.edu.pl (E.N.); katarzyna.przybylowicz@uwm.edu.pl (K.E.P.); 2 Department of Internal Medicine, School of Medicine, Collegium Medicum, University of Warmia and Mazury, 10-900 Olsztyn, Poland; tarlukowicz@wss.olsztyn.pl

**Keywords:** colorectal cancer, cancer, risk factors, symptoms, epidemiology

## Abstract

**Simple Summary:**

According to the available data, colorectal cancer (CRC) is one of the most common malignant neoplasms. Depending on the location, type of cancer or gender, it is ranked 2nd to 4th in terms of incidence in the world. CRC, year by year, shows an increasing tendency in terms of both morbidity and deaths. Many factors may be responsible for the development of this disease, including genetic and environmental factors. Considering all the aspects, we made efforts to systematize the available literature data in terms of epidemiology, risk factors, type and nature of symptoms, development stages, available and future diagnosis of colorectal cancer.

**Abstract:**

This review article contains a concise consideration of genetic and environmental risk factors for colorectal cancer. Known risk factors associated with colorectal cancer include familial and hereditary factors and lifestyle-related and ecological factors. Lifestyle factors are significant because of the potential for improving our understanding of the disease. Physical inactivity, obesity, smoking and alcohol consumption can also be addressed through therapeutic interventions. We also made efforts to systematize available literature and data on epidemiology, diagnosis, type and nature of symptoms and disease stages. Further study of colorectal cancer and progress made globally is crucial to inform future strategies in controlling the disease’s burden through population-based preventative initiatives.

## 1. Introduction

The most common cancer diagnosed in both sexes is lung cancer (11.6% of the total cases), followed by breast cancer in women (11.6%) and prostate cancer in men (7.1%). Colorectal cancer (CRC) is third in terms of recognition (6.1%) and second in terms of mortality (9.2%). It is estimated that by the year 2035, the total number of deaths from rectal and colon cancer will increase by 60% and 71.5%, respectively [1]. These figures may differ from country to country depending on the degree of economic development. Therefore, the disease is widely recognized as a marker of the country’s socioeconomic development [2]. The increase in morbidity is also influenced by lifestyle, body fatness and dietary patterns [3]. There is convincing evidence that physical activity has a protective effect. The risk of developing the disease is increased by more frequent red and processed meat and alcohol drinks [2,4]. The progress of civilization and economic development, apart from improving socioeconomic conditions, also causes a change in dietary patterns, referred to as the westernization of the lifestyle. This means higher consumption of animal fats, processed meats, refined grains or sweets, a low supply of dietary fibers, fruits, vegetables and low physical activity. The occurrence of overweight or obesity is often the result of such a lifestyle [5]. Overweight and obesity are associated with an increased risk of many civilization diseases. Visceral obesity has been reported to adversely affect the prognosis of CRC in men [6]. About a quarter of a contributor to genetic predisposition. The development time of CRC usually lasts from several to several years; therefore, it is very important to diagnose it early in developing the disease. Based on follow-up examinations and nutrition prevention based on a balanced diet, secondary prevention is also important [7].

Considering all the aspects, we made efforts to systematize the available literature data in terms of epidemiology, risk factors, type and nature of symptoms, stages of development and available diagnosis of colorectal cancer.

## 2. Epidemiology

**Figure 1:**
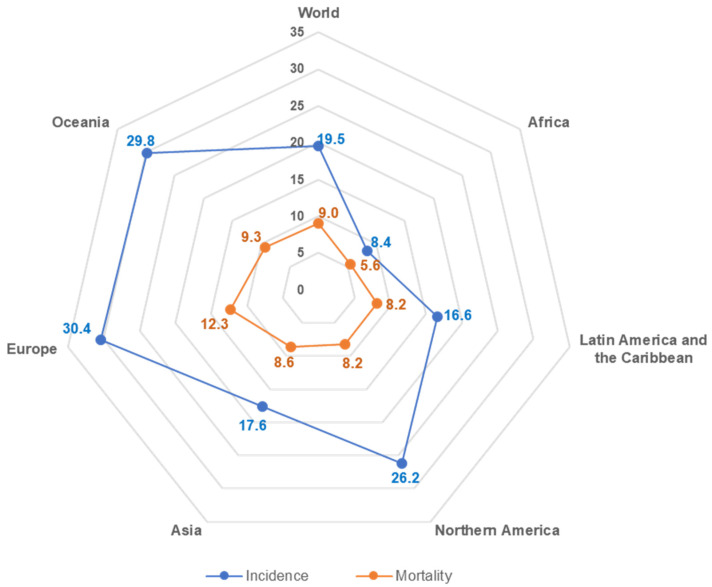
Standardized incidence and mortality rates for CRC for both sexes in 2020, per 100,000.

Colorectal cancer is the third most popular occurring cancer in men and the second most commonly occurring cancer in women. There were over 1.9 million new cases in 2020 [3,8]. Colorectal cancer is the second most common cause of death from cancer, estimated to be responsible for almost 935,000 cancer deaths [3]. Globally it is one of the cancers whose incidence is increasing comprising 11% of all cancer diagnoses [9]. According to GLOBOCAN 2020 data there is a broad geographic variation in CRC incidence and mortality among various countries of the world (Figure 1) [10,11]. It has been recognized that the most significant increase in CRC incidence and mortality occurs in medium and high human development index (HDI) countries that are adopting the “western” way of life [9,10]. Developed countries are at the highest risk of colon cancer. Obesity, sedentary lifestyle, red meat consumption, alcohol and tobacco are considered the driving factors behind the growth of CRC [3,8,9,10,11]. Therefore, colorectal cancer is a disease of developed countries with a western lifestyle [12,13]. Factors that influence life expectancy, including health-related behaviors (smoking, obesity and exercise) and social factors (education, income and government expenditure on health), profoundly impact cancer development. Life expectancy levels must be considered when developing strategies to prevent and treat cancer [12,13]. Interesting data comes from study conducted that in 2007 to 2016, 2006 to 2015 or 2005 to 2014, depending on the data’s availability, colon cancer incidence increased in 10 of 36 countries analyzed (all in Asia or Europe); India had the most significant increase, followed by Poland [3,11]. All 10 of these countries have medium to high (HDI) scores. Six countries had a decrease in colon cancer incidence; these countries had the highest HDI scores; the United States had the most significant reduction, followed by Israel. Seven countries (including all countries from Northern America) had a decrease in incidence among persons older than 50 [9,10,11]. Eight countries had an increase in colon cancer incidence among persons younger than 50 years, including the United Kingdom and India. Countries with a decreased or stable incidence among persons 50 years or older but a significant increase in persons younger than 50 years included Germany, Australia, the United States, Sweden, Canada and the United Kingdom [8,9,10,11]. The decline in the incidence of CRC was recorded only in Italy among people under the age of 50. Among women, 12 of 36 countries (all from Asia and Europe) had an increase in colon cancer incidence, and seven countries had a decrease; India had the most significant growth, followed by Slovenia [9,10]. Many works have attracted attention that colorectal cancer survival depends on the stage at which it is diagnosed, with later-stage diagnosis having lower survival [2,3,8,9]. The five-year survival rate is 90 percent for colorectal cancers diagnosed at an early stage compared with 13 percent for those diagnosed later. At age 0–74, the cumulative risk of dying from colon cancer is 0.65% among men and 0.45% among women [3,8,11]. Age-standardized (world) mortality rates per 100,000 of CRC in both sexes is 8.9 [3].

In recent years, the global burden of CRC will increase by 60%, to over 2.2 million new cases and 1.1 million deaths by 2030. Such a significant increase will be the result of economic development, an economic transformation consisting in the transition from low-to-medium-HDI nations and generational changes in developed countries. Many research studies emphasize that this increase is also the result of environmental changes, such as a more sedentary lifestyle, abnormal bony weight (obesity), consumption of highly processed food, alcohol, red meat consumption and an increase in overall life expectancy [2,9,10]. With the best scientific understanding in mind, an updated study of the current patterns and temporal trends of CRC from a global perspective is critical to developing future strategies for prevention and treatment programs to reduce disease incidence. Many research works emphasize the need to allocate resources for health education focused on CRC risk factors and to formulate screening programs using the latest scientific reports in the aspect of public health.

## 3. Risk Factors

**Figure 2:**
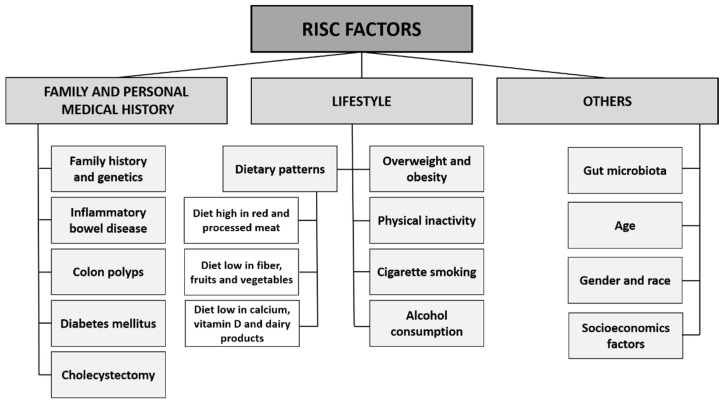
The main risk factors associated with colorectal cancer.

Multiple factors have been implicated in the development of colorectal cancer (Figure 2). It was demonstrated that individuals are at increased risk for CRC if they (or their relatives) have had cancer, a history of colon polyps, inflammatory bowel diseases, diabetes mellitus or cholecystectomy. Lifestyle factors also play important roles in CRC etiology. The evidence shows that overweight and obesity, physical inactivity, cigarette smoking, alcohol consumption and inappropriate dietary patterns (a diet low in fiber, fruits, vegetables, calcium and dietary products and high in red and processed meat) increase CRC risk. In addition, gut microbiome, age, gender and race and socioeconomical status are known to influence colorectal cancer risk.

### 3.1. Family and Personal Medical History

#### 3.1.1. Family History and Genetics

A family history of colorectal cancer significantly increased the risk of developing colorectal cancer. This phenomenon shares both inherited genetic predisposition and lifestyle factors. The information relevant for future colorectal cancer occurrence, among other, include: (i) the generational distance of the relatives to the individuals at risk; (ii) the age at which the first-degree relatives developed colorectal cancer; (iii) the number of family members diagnosed with colorectal cancer; (iv) family co-occurrence of other neoplasms (e.g., endometrial, ovarian and urinary tract, pancreatic) and (v) personal history of cancer. Previous studies indicated that people with one affected first-degree relatives (parents, siblings and children) have, on average, two times higher risk of CRC in comparison to those with no family history. The risk of CRC development is significantly higher if a relative is diagnosed before the age of 60. Moreover, a higher number of affected relatives (not only first-degree but also second- and third-degree) also increases the disease risk [14,15,16,17].

It is estimated that 2–8% of colorectal cancer cases arise as a result of inherited syndromes. The two most common hereditary syndromes that predispose for colorectal cancer development are hereditary nonpolyposis colorectal cancer (HNPCC), also known as Lynch syndrome, and familial adenomatous polyposis coli (FAP). HNPCC is an autosomal dominant disease caused by mutations in genes known as mismatch repair errors. Proteins encoded by these genes are responsible for reaper errors in DNA that occur during cell division. Most cases of HNPCC are associated with mutations in MLH1 and MSH2 genes. However, there are several other genes mutations in which give rise to HNPCC (e.g., MSH6, MLH3, TGBR2, PMS1 and PMS2). Patients with HNPCC have about 20% risk of developing CRC by the age of 50 and about 80% risk of developing CRC by the age of 85 [15,18,19,20,21].

Similar to HNPCC, FAP also presents an autosomal dominant pattern of heredity. It is caused by adenomatous polyposis coli (APC) gene defects. APC is classified as a tumor suppressor gene. It encodes a protein that plays a significant role in regulating DNA replication and cell division. Individuals with FAP start to develop hundreds or even thousands of colon polyps in their mid-teens and, with high-probability, most of these colon polyps evolve into cancer. It is assumed that almost all patients with the earlier unrecognized and untreated FAP syndrome will be diagnosed with colorectal cancer before the age of 35–40 [19,20,21,22].

The increased risk of CRC development is also linked with the occurrence of Peutz-Jeghers syndrome, Juvenile polyposis syndrome, PTEN hamartoma tumors syndrome and MUTYH-associated polyposis (MAP) [21].

#### 3.1.2. Inflammatory Bowel Disease (Crohn’s Disease; Ulcerative Colitis)

Inflammatory bowel disease (IBD) is ranked as the third-highest risk condition for the development of colorectal cancer, behind HNPCC and FAP. IBD is a group of chronic and incurable diseases, which affect the immune system of the gastrointestinal tract and, in consequence, lead to the development of uncontrolled inflammation. The two major forms of IBD are Crohn’s disease and ulcerative colitis. The etiology of IBD is unknown, it is considered that the development of IBD is a result of interactions between genetic, immunological and environmental factors [20,23]. Due to the fact that chronic inflammation promotes tumor growth and progression, individuals with IBD have about 2–6 times higher risk of developing CRC in comparison to healthy individuals. The risk of CRC increases with the duration of IBD and the anatomic extent and severity of the disease [14,24,25].

#### 3.1.3. Colon Polyps

**Figure 3:**
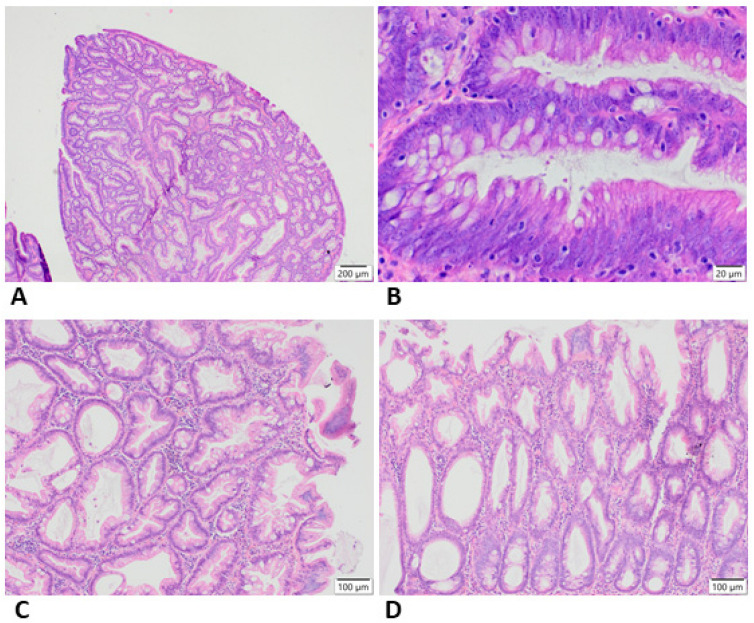
Representative histopathological appearance of adenomatous (**A**,**B**) and serrated (**C**,**D**) changes in the colon.

Colon polyps (precancerous neoplastic lesions) are defined as an abnormal growth of tissue projecting from a mucous layer of the colon. They are histologically classified into two main categories: non-neoplastic (hamartomatous, hyperplastic and inflammatory polyps) and neoplastic (adenomatous, Figure 3). The adenomatous polyps are of great importance because they harbor the potential to become malignant. It is estimated that about 95% of colorectal cancer is developed from adenomatous polyps. Despite the fact that almost all cancer arises from adenomas, it is estimated that only about 5% of polyps progress to colorectal cancer [16,26]. The period for the transition of adenomatous polyps into invasive adenocarcinoma is 5–15 years and the risk of malignant transformation increases with polyp size, degree of dysplasia and the age of individuals. Polyps greater than 1–2 cm in diameter, a high degree of dysplasia and increasing age are unfavorable prognostic factors. Due to the fact that approximately 40% of people at the age of 50 or older have one or more adenomatous polyps, it is of great importance to identify these polyps and remove them prior to cancer transition [14,26].

#### 3.1.4. Diabetes Mellitus

Diabetes mellitus is a metabolic disorder characterized by chronic hyperglycemia, which results from defects in insulin secretion and/or action. It is estimated that around 460 million people globally are currently suffering from diabetes and the number will continue to grow. Epidemiological data indicate that diabetes is an independent risk factor for several gastrointestinal cancers, including colorectal cancer [27,28]. Individuals with type 2 diabetes have about two-three times greater risk of developing colorectal cancer in comparison to the non-diabetic population [29,30]. The development of colorectal cancer is thought to be related to an increase in insulin concentration and an inflammatory condition associated with the disease. Hyperinsulinemia may contribute to colorectal cancerogenesis directly by stimulating colonic cell proliferation and indirectly by increasing the level of insulin-like growth factor 1 (IGF-1). IGF-1 is a mitogenic factor that enhances cell growth and decreases cell death [27,31]. Moreover, chronic inflammation associated with diabetes favors carcinogenesis, malignant transformation, tumor growth, invasion and metastasis through the action of proinflammatory cytokines, such as tumor necrosis factor-alpha (TNF-α) and interleukin-6 (Il-6) [31,32].

#### 3.1.5. Cholecystectomy

The possible association between cholecystectomy, the surgical removal of the gallbladder from the body and subsequent colorectal cancer incidence has still not been firmly established or refuted. The results from some studies indicated an increased risk of CRC development after cholecystectomy [33,34,35], whereas others have reported no increased risk [36,37,38]. The possible increased risk of CRC after cholecystectomy is thought to be associated with changes in the secretion and composition of bile acids. Under physiological conditions bile acids are released periodically in response to food intake. In the absence of a gallbladder, there is a continuous flow of bile to the intestine, which results in increased bacterial biotransformation of bile acids into secondary bile acids. The secondary bile acids have the potential to generate reactive oxygen and nitrogen species, disturb the cell membrane and induce DNA damage and apoptosis in the colonic mucosa cells, which increase the risk of developing colon carcinomas [39,40].

### 3.2. Lifestyle

#### 3.2.1. Dietary Patterns

- Diet high in red and processed meat

According to the International Agency for Research on Cancer Group, red meat and processed meat were classified as probably carcinogenic to humans (Group 2A) and carcinogenic to humans (Group 1), respectively. Red meat is defined as the meat derived from the muscle of farm animals (e.g., beef, lamb, game and pork). Processed meat refers to the meat that has been preserved by curing, salting, smoking or adding chemical preservatives in order to improve favor or extend the shelf life. Studies have shown that regular consumption of red and processed meat is an important risk factor for the development of colorectal cancer [20,41]. It is estimated that the risk of CRC may increase by about 17% for every 100 grams portion of red meat and by approximately 18% for every 50 grams of processed meat eaten daily [42,43,44]. The exact mechanism by which consumption of red and processed meat may contribute to the development of colorectal cancer is still under investigation. Several factors that are believed to influence the occurrence of cancer are heterocyclic amines (HACs), polycyclic aromatic hydrocarbons (PAHs) and N-nitroso compounds (NOCs)—harmful substances that may be produced during high-temperature or open-fire cooking of meat (e.g., pan-frying, grilling and roasting). HACs are formed during the specific reaction of free amino acids, carbohydrates and creatinine or creatine (substances found in muscle). PAHs, in turn, are formed when fat and juice from meat come into contact with open flames. The smoke that contains PAHs attaches to the surface of the cooked meat. HACs and PAHs are considered genotoxic substances that have the potential to cause point mutations (deletions, insertions and substitutions) and, in consequence, initiate the process of carcinogenesis. Similarly, NOCs (nitrosamine and nitrosamide) are potent carcinogenic agents that can react with DNA. These substances are synthesized from amines or amides and oxides of nitrogen (nitrites or nitrates, i.e., substances used as a food additive to inhibit the growth of bacteria and gives the meat the desirable cured) during high-heat cooking of processed meat [45,46]. The other factor that is believed to contribute to the malignant transformation of colon cells is heme, an iron-containing porphyrin presents in large amounts in red meat. It was demonstrated that heme increases oxidative stress and induce lipid peroxidation of intestinal cells. Reactive oxygen species contribute to DNA damage and gene mutations. Reactive lipid peroxides, in turn, exert a cytotoxic effect on epithelial cells. The damage of the cell surface results in hyperproliferation of the cells and leads to epithelial hyperplasia, which may evolve to dysplasia and cancer. In addition, heme irons stimulate the endogenous formation of the above-mentioned NOCs and induce alternation in the gut microbiota leading to a state of dysbiosis [5,41,44]. It should be also emphasized that consumption of high-fat red and processed meat contributes to obesity, insulin resistance and an increase of bile acid secretion, which acts as an aggressive surfactant for the mucosa and increase the risk of developing colorectal cancer.

- Diet low in fiber, fruits and vegetables

It was shown that the high consumption of dietary fiber could reduce the risk of colorectal cancer development by up to 50% [14]. However, currently available results of epidemiologic studies not unequivocally support the protective effects of fiber against CRC and the precise mechanism of anticancer fiber action has not been clearly established. The potential mechanism of the protective effect of fiber consumption on CRC development includes: (i) reduction of transit time for stool throughout the colon and, in consequence, reduction of contact between potential carcinogenic substances and colonic epithelium, (ii) increase in the amount of water in fecal content and thus dilution of carcinogens and procarcinogens present in fecal, (iii) binding sterols and bile acids metabolites, which may be implicated in carcinogenesis, and (iv) stimulation the growth of beneficial gut microbiota, which, in turn, ferment fiber and produce short-chain fatty acids—substances suggested to exert tumor-suppressive effects. Therefore, dietary guidelines recommend people consume at least 20–30 g of fiber per day [5,16,17,41]. Naturally great sources of fiber are fruits and vegetables. In addition to fiber intake, consumption of fruits and vegetables provides a large number of bioactive compounds, such as vitamins, minerals, folate, plant sterols and protease inhibitors. Many of these compounds exhibit potent antioxidant and anti-inflammatory properties, which could inhibit DNA and cellular damage. The results from several studies demonstrated that a high intake of fruits and vegetables may be linked with a lower CRC risk development [17,24,41].

- Diet low in calcium, vitamin D and dairy products

According to the World Cancer Research Fund/American Institute for Cancer Research [41], the high consumption of dairy products (in particular milk) is probably inversely associated with the risk of developing colorectal cancer. The suggested protective effect of dairy products has been largely attributed to their content of calcium. It was demonstrated that calcium binds secondary bile acids and fatty acids diminishing their ability to modify intestinal mucosa and, in consequence, limiting their carcinogenic potential. Moreover, calcium was found to inhibit proliferation and to induce apoptosis of tumor cells and reduce distinct patterns of mutation in proto-oncogene KRAS [5,41,47]. In addition to calcium, the other milk component, i.e., vitamin D is also suggested to play a beneficial role against CRC development. The roles of vitamin D and calcium are closely related since the primary function of vitamin D is the maintenance of calcium homeostasis by enhancing its intestinal absorption. It is hypothesized that the anticancer effect of vitamin D may be a result of the increased level of serum calcium concentration. It should be emphasized, however, that vitamin D exerts many other physiological functions that may play an important part in cancer control. The results of the studies showed that vitamin D alters the expression of a variety of genes involved in the regulation of growth, proliferation, differentiation and apoptosis of epithelial cells. Moreover, it exhibits anti-inflammatory action, improved immune function and inhibits angiogenesis [48,49]. Due to the fact that the major source of vitamin D for humans is skin exposure to sunlight, there are some studies to determine if the distribution of colorectal cancer incidence depends on amounts of natural light. It was demonstrated the colorectal cancer mortality rates were higher in the northern regions of the United States and Europe. It is assumed that people who live at higher latitudes are exposed to less amount of solar ultraviolet-B dose, synthesize less vitamin D and therefore have a higher risk of developing and die from colorectal cancer [48]. On the other hand, the results of the study performed in Norway showed that there is no significant north–south gradient for the death rate for colon cancer. However, the survival rate of colon cancer depended on the season of diagnosis and was the lowest in the cancers diagnosed in the autumn. Recent meta-analyses of prospective cohorts demonstrated that, regardless of geographic location, higher serum vitamin D level was related to a statistically significant, substantially lower colorectal cancer risk in women and non-statistically significant lower risk in men [50]. According to World Cancer Research Fund/American Institute for Cancer Research [41], the evidence for vitamin D was limited and there is a need to perform research assessing the anticancer activity of vitamin D.

#### 3.2.2. Overweight and Obesity

A condition of abnormal or excessive fat accumulation (overweight and obesity) is a convincing risk factor for the development of colorectal cancer. Overweight/obese men and women have about 50% and 20% greater risk of developing colorectal cancer in comparison to people with normal weight, respectively. It is estimated that an overall CRC risk increase by 3% for every five kilograms of weight gain [17,20]. The mechanisms underlying the induction of carcinogenesis in overweight/obese people are not fully understood and still under intense study. Adipose tissue is an endocrine organ that plays a crucial role in the regulation of energy intake and inflammatory response. It was found that abnormal or excessive fat accumulation causes alternations in adipose tissue hormone and cytokine secretions. Adipose tissue of overweight/obese people release more factors (e.g., leptin, resistin, TNF-α, IL-1, IL-6, IL-7 and IL-8), which are known to exhibit mitogenic effects on epithelial cells, inhibit apoptosis of the cells, promote oxidative stress, suppress immune response and reduce the activity of IGF-1 axis and have been associated with cancer development and progression [5,41,51].

#### 3.2.3. Physical Inactivity

Epidemiological data indicate that an increasing colorectal cancer incidence in developed and developing countries may be the result of a sedentary lifestyle. It is estimated that physically inactive people have up to 50% higher risk of developing colorectal cancer in comparison to the most physically active ones [17,52]. Regular physical exercises have been shown to improve immune system function, reduce inflammation, reduce stress, optimize metabolic rate, help regulate hormone level and prevent obesity and, as a result, may help protect against cancer development [47].

#### 3.2.4. Cigarette Smoking

Tobacco smoke is an established risk factor for the development of many types of cancer, including colorectal cancer. The results of the studies indicated that people who smoke cigarettes have to 2–3-fold increase risk for developing CRC in comparison to non-smokers and the risk increases with dose and duration of exposure [31]. In addition, it is considered that cigarette smoking is attributed to up to 12% of colorectal cancer deaths [16]. Tobacco smoke contains a mixture of thousand chemicals, over 60 of which are well-established carcinogens (e.g., N-nitrosamines, polycyclic aromatic hydrocarbons, aromatic amines, aldehydes and metals) that are known to damage DNA. Mutations in colorectal epithelial cells may lead to polyposis development, which, in turn, may transit into invasive adenocarcinoma [53].

#### 3.2.5. Alcohol Consumption

Alcohol intake is one of the major contributors to colorectal cancer development. It is estimated that the consumption of 2–3 drinks daily increases the risk of CRC by about 20%, whereas drinking more than three alcoholic beverages increases this risk by about 40% [17,20]. Individuals who are used to drink four and more drinks every day increase their chance of developing colorectal cancer for up to 52% [54]. To date, the various mechanism by which alcohol may induce carcinogenesis have been proposed. They include the production of reactive oxygen species and nitrogen species (during the oxidative metabolism of ethanol), production of mutagenic acetaldehyde (the first metabolite of ethanol), depletion of S-adenosylmethionine (epigenetic alternations), inactivation of the tumor suppressor genes, hormonal imbalance, reduction in folate concentration and impairment of retinoic acid metabolism [47,55].

### 3.3. Others

#### 3.3.1. Gut Microbiota

Recently, a growing number of studies indicated that gut microbiota may be a key factor that contributed to the development of many pathological processes, including cancer. The gut microbiota (microbiome) comprises a large population of diverse microorganisms (bacteria, viruses, fungi and protozoa) inhabiting the gastrointestinal tract of humans. In healthy people, the microbiome is involved in nutrient metabolism and absorption, drug metabolism and elimination of xenobiotics. In addition, normal gut microbiota participates in the maintenance of intestinal barrier integrity, protects against pathogens and plays an important role in immunomodulation. According to the latest research that explored the microbiome of the individuals with colorectal cancer, alternation in the composition and functionality of the normal gut microbiota may lead to initiation, promotion and progression of this cancer. It was demonstrated that toxic metabolites of bacteria cause DNA damage, affect cell cycles, stimulate immune response and lead to disturbance of the intestinal barrier function. As a result, impaired intestinal microbiota homeostasis contributes to the development of the microenvironment favorable to develop colorectal cancer [56,57,58,59,60].

#### 3.3.2. Age

Due to the fact that about 90% of all new cases of colorectal cancer occurring in individuals over 50 years old, older age is considered to be one of the most significant factors influencing the risk of developing colorectal cancer [14,16]. It is estimated that people after the age of 65 have about three times greater risk to develop colorectal cancer in comparison to those at the age of 50–64 and about 30 times greater risk than people at the age of 25–49 [20]. The average age at diagnosis is 68 and 72 years old for men and women, respectively. The fact that colorectal cancer is the age-related disease is particularly evident in the developed countries where the rate of colorectal cancer is the highest. The number of colorectal cancer incidence in these countries is associated, among others, with longer life expectancy and, in consequence, increase number of old people in the population [61]. It should be emphasized, however, that the results of the newest studies indicated that the incidences of colorectal cancer rise among young adults (20–49 years old) in the United States and Europe [62,63]. Currently, it is recommended to begin screening for colorectal cancer in adults aged more than 50 years. According to the authors of the studies, if the mentioned trend continues, screening guidelines should be reconsidered.

#### 3.3.3. Gender and Race

According to the American Cancer Society, men have about 30% higher risk of developing colorectal cancer in comparison to women. In addition, men who are diagnosed with colorectal cancer have a worse prognosis and approximately 40% higher mortality compared to women [17]. On the other hand, women are more prone to develop right-sided colon cancer, which is often diagnosed at a more advanced stage and seemed more aggressive than left-sided tumors [64,65]. The reasons for sex disparity are not fully understood, it is considered that they may be related to the differences in the exposure to risk factors (e.g., alcohol and tobacco), dietary patterns and sex hormones [17,47].

The incidence of colorectal cancer varied substantially by race also. The non-Hispanic Black individuals experience one of the highest incidence rates of all racial groups. It is estimated that colorectal cancer incidence rate in non-Hispanic Blacks is approximately 50% higher than in Asians/Pacific Islanders and about 20% higher than in non-Hispanic Whites [17,66].

#### 3.3.4. Socioeconomics Factors

It is believed that people with low socioeconomic status (SES) generally have a higher risk of developing cancer than those with high SES. This may be explained in part by limited access to health care services and high-quality treatment resources and unhealthy dietary habits, sedentary lifestyle and smoking in the low socioeconomic status population [16,67]. It should be emphasized, however, that the results concerning the association of SES with the incidences of colorectal cancer are inconsistent. In North America, people with low SES exhibited a higher incidence of colorectal cancer in comparison to people with high SES, contrary, in Europe, high SES groups often show a higher risk of developing colorectal cancer. Therefore, there is a need to perform additional studies in order to establish the impact of socioeconomic status on colorectal cancer occurrence [68].

## 4. Development Factors

**Figure 4:**
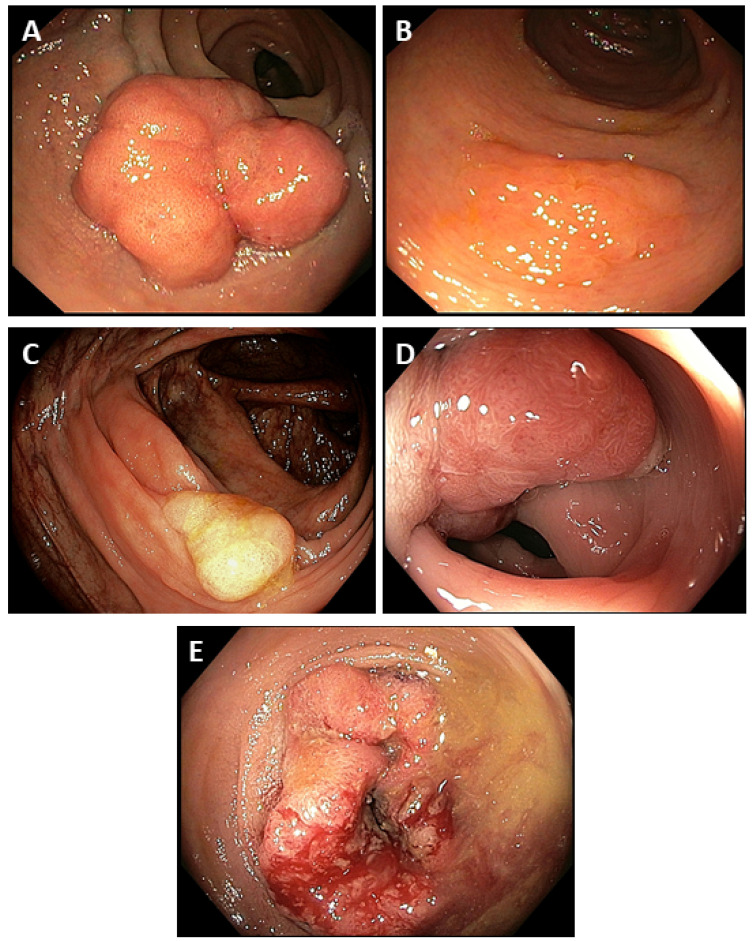
Selected endoscopic images of adenomas and CRC at different stages. (**A**)—Tubular adenoma; (**B**)—tubulo-villous adenoma; (**C**)—sedentary serrated adenoma (SSA) without dysplasia; (**D**)—tubular adenocarcinoma, grade 1 and (**E**)—tubular adenocarcinoma, grade 2.

**Figure 5:**
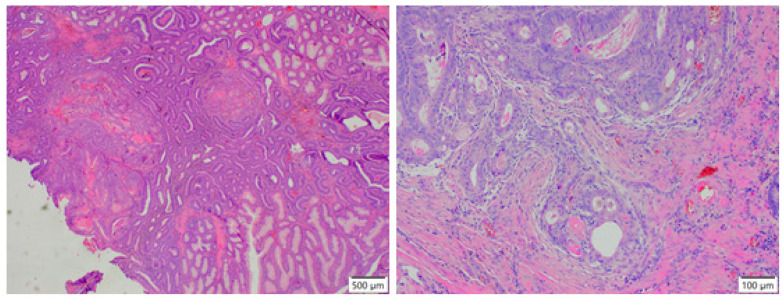
Representative histopathological appearance of adenocarcinoma in the colon.

The formation of CRC consists of the stages of initiation, promotion and progression. Initiation involves irreversible genetic damage that predisposes the intestinal mucosa’s affected epithelial cells to subsequent neoplastic transformation [69]. In the promotion phase, the initiated cells multiply, generating abnormal growth (cancer). In contrast, benign cancer cells turn into malignant ones during the progression stage and acquire aggressive features and metastatic potential [24]. A crucial part of most CRC carcinogenesis steps is the presence of a benign precursor lesion, defined as a polyp (defined as abnormal growth on the colon mucosa growing in its lumen). Another type of lesions identified in the large intestine lumen is adenomatous polyps (adenomas, Figure 4) and serrated polyps, which are the direct precursors of most cancers [20,70]. Advanced adenomas (≥1 cm in diameter) with or without diversity have a significantly higher risk of cancer progression (from 30 to 50%) than non-advanced adenomas (1%). Moreover, advanced adenomas characterizing the higher transition rates to cancer, increasing with age [71,72]. The other changes in the gut wall, such as polished polyps, represent a group of heterogeneous lesions, which include: hyperplastic polyp, traditional serrated adenoma, sessile serrated adenoma and mixed polyp [73]. They combine the toothed morphological appearance of hyperplastic polyps and dysplastic features of adenomas, and these changes are precursors to approximately 10–15% of sporadic CRC. However, the most common lesion present in the gut is a hyperplastic polyp (80–90%) [53]. The research showed that the hyperplastic polyps (especially large and/or in the proximal colon) could pass in the CRC as part of a serrated pathway through traditional serrated adenoma or serrated sessile adenoma [74]. Undoubtedly, the process of CRC carcinogenesis is quite slow, starting with a slight inflammation, then through the development of adenomatous polyps in the epithelium, and finally, the development of adenocarcinoma (Figure 5) [75]. Moreover, the process is driven by the accumulation of mutations and genetic changes and takes 10–15 years, but maybe faster in some conditions, e.g., in patients with Lynch syndrome [76].

About 20% of CRC is associated with hereditary syndromes such as familial adenomatous polyposis (FAP), Lynch syndrome (HNPCC), mutation-related polyposis MUTYH (MAP) and hamartomatous polyposis syndromes (Peutz-Jeghers, juvenile polyposis and Cowden disease) [51,77,78]. In the case of HNPCC, one allele of the DNA repair gene, while in FAP, one allele of the adenomatous polyposis tumor suppressor (APC) gene is inactivated by the germline [77]. Moreover, about 80% of people with FAP have an affected parent, including about 20% of cases are de novo mutations. It is estimated that 95% of people with FAP develop adenoma as early as 35 [78]. A colectomy is then indicated, recommended when more than 20–30 adenomas have been designed, or multiple adenomas with advanced histology have developed. HNPCC is associated with pathogenic variants of the MLH1, MSH2, MSH6, PMS2 and EPCAM genes, and is also characterized by an increased risk of CRC, the pathological feature of which is the presence of mucinous adenocarcinoma (lifetime risk at 52–82%, mean age at diagnosis 44–61 years) [23,79]. Moreover, this neoplasm may predispose to asynchronous or metachronous colorectal neoplasm [68].

CRC may also arise in the inflammatory pathway in patients with inflammatory bowel disease, particularly ulcerative colitis. In these patients, from the absence of dysplasia, through dysplasia for an indefinite period, low-grade dysplasia, to high-grade changes towards neoplastic transformation, finally, CRC occurs [24].

The most commonly affected genes in the CIN pathway (the chromosomal instability pathway) are APC, p53 and K-ras, which are responsible for the adenocarcinoma sequence pathway. Changes within these genes lead to mutational activation of oncogenes or inactivation of tumor suppressors, which consequently causes malignant transformation. CIN pathway is responsible for 70–85% of all CRC cases [80,81,82]. Apart from the mechanisms related to chromosomal instability (CIN) and microsatellite instability (MSI—microsatellite instability), a third one should be mentioned, related to the methylator phenotype (CIMP—CpG island methylator phenotype) [83,84]. It is associated with hypermethylation of numerous gene promoters (including MLH1), the V300E mutation in the BRAF gene, and loss of TP53 and p16 functions. These disorders cause silencing of suppressor genes and thus disturbances in the MMR system’s functioning, the occurrence of MSI and the state of hypermutation. This mechanism is most often observed in the development of serrated architectural lesions, most often in women in the colon’s proximal part [85].

As mentioned above, CRC is a non-homogeneous disease entity. Individual cases differ in location, degree of histological malignancy or the type of neoplasm. However, the most exciting thing is the multilevel molecular complexity. The consensus developed in 2015 by the CRC Subtyping Consortium identified four molecular subtypes of colorectal cancer (CMS): CMS1—MSI-immune activation, CMS2—canonical, CMS3—metabolic and CMS4—mesenchymal. The classification is of practical importance: individual subtypes differ in their clinical course and respond differently to chemotherapeutic and biological treatment. This may determine the selection of the optimal, individualized therapeutic strategy for each patient and is also a helpful predictive and prognostic tool. Perhaps the most promising is the application of these phenomena to molecular screening for colorectal cancer [86].

In 10% of all colorectal cancers, serrated adenocarcinomas develop by replacing adenomatous polyps with serrated polyps on the so-called serrated lesion pathway, showing the presence of the BRAF mutation and epigenetic silencing of various genes, but without APC gene involvement, as is the case in other pathways. Another mechanism leading to CRC is microsatellite instability (MSI), caused by the disruption of DNA repair genes [44,80,87]. The inherited genetic predisposition and exposure to environmental factors may work together to form adenomas and cancer based on synergy [44]. However, most CRCs are sporadic, meaning that patients do not have a genetic burden, and the development of this type of cancer is linked to lifestyle and environmental factors. Moreover, long-term exposure to carcinogens may promote oxidative stress. Oxidative stress can be increasing DNA damage by generating sequential accumulation of somatic mutations, leading to genome instability [44].

## 5. Symptoms

CRC may be suspected when some of the lower gastrointestinal (GI) symptoms are present. National Institute for Health and Professional Excellence has published guidelines on which basis health practitioners may identify patients with a high CRC probability. Suspected CRC recognition and referral for future diagnosis are related to the occurrence of rectal bleeding, abdominal mass, abdominal pain, change in bowel habit, unexplained weight loss and iron-deficiency anemia [88]. However, some non-site-specific symptoms, such as unexplained appetite loss and deep vein thrombosis, should be mentioned. For these symptoms, an assessment for additional symptoms, signs or findings may help clarify which cancer is most likely to be carried out and offer urgent investigation or a suspected cancer pathway referral [89].

In some research the usefulness of symptoms of detecting CRC has been evaluated. They present single signs or symptoms that have low utility (sensitivity and specificity) for the CRC diagnosis. Moreover, both positive and negative likelihood ratios (PLR and NLR) confirm the presence or lack of symptoms does not significantly modify the probability of CRC detection [90,91,92]. Nevertheless, in clinical practice, according to many guidelines, colonoscopy is performed in patients with bowel signs and symptoms suspected of CRC [88]. However, some studies suggest that co-occurrence of some symptoms may enhance the diagnostic sensitivity and specificity for colorectal cancer. [90,91], e.g., the presence of a palpable abdominal mass on examination and a report of dark red rectal bleeding [90] or rectal bleeding and weight loss and change in bowel habit [92].

In the context of CRC treatment, patients who have been diagnosed before they had symptoms of CRC (or these were the first symptoms) and the disease was detected at an early stage have a much better prognosis. For this reason, all alarming symptoms that may suggest CRC should encourage the patient to see a doctor urgently and have colorectal diagnostic tests done [93].

## 6. Diagnostic

For individuals suspected of having CRC, primary care physicians should carry out a physical examination of the abdomen and analyze the health history to diagnose. The suspicion of CRC on physical examination and subject examination indicates that the patient is referred to a gastroenterology clinic. During the visit, the doctor should consult patients in terms of family history, consider assessing risk factors, and then choose an appropriate optical and/or imaging diagnosis method. Another pathway for detecting CRC is various screening programs (pilot, opportunistic or organized) placed worldwide [94]. Despite a higher number of programs, the participation ranged from 16.1% to 68.2% [95]. The programs mostly include individuals aged 50–75 years with wide variations in screening practices depending on the protocols resulting from the study stage, colonoscopy capacities and financial resources. Screening programs are implemented more frequently in Western countries with higher CRC prevalence with a different type of test. Most of screening diagnostic methods include fecal immunochemical test for hemoglobin (FIT), guaiac fecal occult blood test (gFOBT), (optical) colonoscopy (OC), flexible sigmoidoscopy (FS) and digital rectal exam (DRE) [94].

Assessment of family history of cancer in first (FDR), second (SDR) and third-degree relatives is essential to obtain detailed information in the diagnosis process [96]. Taken information should include relative consanguinity, age at cancer diagnosis, current age or age and cause of death, type of cancer, its medical case history and ethnicity. Research provides that the risk of CRC is the highest, along with patients with FDRs with CRC [96]. Additionally, the number and degree of relatives determine the screening pathway for CRC diagnosis. In case of one FDR with CRC or more than one FDRs with advanced adenoma was reported, the patient is examined by colonoscopy every 5–10 years or FIT every 1–2 year begins at the age of 40–50 years or 10 years earlier than FDR age of diagnosis. In case of more than one FDRs or SDRs with CRC or polyps and more than two FDRs with CRC is reported in the patients’ family history, the colonoscopy should be done every 5 years beginning at the age of 40 or 10 years earlier than the age of FDR diagnosis [97]. Other information should include potential determinants such as non-paternity or born resulting from sperm/egg donors. Suppose suspected CRC, syndromes and other syndrome-specific features are diagnosed in personal or family history (e.g., Lynch syndrome, familial adenomatous polyposis, MUTYH-associated polyposis and hamartomatous polyposis syndromes). In that case, the patient should follow high-risk guidelines, and the surveillance should be started by age 20–25 [98,99]. Additionally, in any patient with suspected colorectal cancer, it is recommended to pay attention to peripheral lymphadenopathy, hepatomegaly, a palpable abdominal tumor and the presence of ascites.

The fecal occult blood test is the first-choice screening test in primary care. However, it has been recommended for their implementation to refer patients with low-risk bowel symptoms but has not been recommended for all symptomatic patients [88]. For CRC screening and detection of occult bleeding, high-sensitivity, guaiac-based (HSgFOBT) or immunochemical-based (FIT) tests are recommended [99]. gFOBT is not specific to human hemoglobin, and some foods or drugs can affect the results of this test; therefore, it requires some restriction to comply before tested [98]. FIT measures the amount of human-specific hemoglobin in a feces sample and is recommended in place of gFOBT for patients with low-risk CRC symptoms. NICE guidelines recommend it for the patient with unexplained changes in bowel habits and iron deficiency anemia (patients aged 60 and over, even in the absence of iron deficiency) for use in primary care or screening for suspected CRC [88]. FIT may help effectively excluded CRC among symptomatic patients [100] and, in conjunction with clinical assessment, may safely and objectively determine individual risk of CRC for further decisions about urgent or routine management [101]. Moreover, FIT is preferable to the gFOBT in terms of the detection rate, positive predictive value and participation rate [102]. Recent meta-analyses confirm that quantitative FIT is highly sensitive for CRC detection [103] and indicated that at a cut-off around 10 μg Hb/g faces has the potential to rule out CRC correctly and decrease colonoscopy rate in 75–80% of symptomatic patients [104]. Recommendation to routinely perform FIT in primary care in individuals with unexplained symptoms but no rectal bleeding who do not meet criteria for suspected CRC is currently not sufficiently evidenced [88]. However, recent research indicates that FIT performs exceptionally well to triage patients with low-risk CRC symptoms [105].

Endoscopy (colonoscopy, sigmoidoscopy and rectoscope) is the basis for a diagnosis of CRC. It allows tumors to be detected, samples to be taken, and the rest of the bowel to be inspected. Flexible sigmoidoscopy allows visualization of the left-side colon and, if necessary and possible, remove polyps. It does not require thorough patient preparation for the examination as colonoscopy and can be performed by physicians and non-physicians [98]. Diagnosis by colonoscopy is the procedure with the highest sensitivity and specificity for the diagnosis of colorectal cancer. Colonoscopy makes it possible to assess the entire large intestine and the terminal part of the small intestine. During the examination, it is possible to take a biopsy and then have the material evaluated histopathologically [106]. High-quality baseline colonoscopy has to meet adequate bowel preparation criteria, complete examination to the cecum, attention to complete polyp excision and performed by a colonoscopist with acceptable adenoma detection rate [107]. Further scheduling of surveillance colonoscopies depends on the results of the number and size of polyps and adenomas detected during baseline colonoscopy [107]. New methods such as artificial intelligence are implemented in colonoscopy to support and improve its effectiveness in detecting and assessing colorectal polyps. A computer-aided diagnostic system (CAD) that uses deep-learning technology can accurately determine polyp histology (from 63.8–71.8% to 82.7–84.2%) and may facilitate endoscopist diagnosis [108]. Additionally, results of deep neural network demonstrated better polyps detection with using narrow-band imaging than white light endoscopy (WLE) (accuracy 95% vs. 74%) and using the two-channel red plus green images than full-color WLE images (74% vs.91%) [109].

As invasive endoscopy tools are the ideal methods for detecting cancer at an earlier curable stage and removing the precancerous adenomas, some non-invasive methods are accessible to the whole visualization colon with good sensitivity and specificity; however, it does not allow biopsy during imaging. Colon capsule endoscopy (CCE) can be used as an alternative to colonoscopy in screening patients at moderate CRC risk when conventional colonoscopy cannot be performed or is contraindicated or rejected by patients. First-generation CCE has low-quality evidence that would deceive a good sensitivity and specificity for detecting CRC polyps and has a good safety profile [110]. However, the sensitivity in the detection of polyps >6 mm and >10 mm increased substantially between the development of its first-generation and second-generation [111], which has a wider angle of view and an adaptive frame rate dependent on the speed of passage of the capsule into the colon [112]. Despite CEE having a good accuracy in detecting polyps and colorectal cancer among high- and middle-risk patients [113], it is not recommended as a first-line screening or diagnostic method for CRC [114].

Computed tomographic colonography (CTC) is a non-invasive, rapid radiographic imagining test. The patient’s preparation for the examination is the same as for colonoscopy, and the examination itself is not very pleasant due to the discomfort caused by the insufflation procedure [106]. High-quality evidence supports its strong recommendation as an acceptable and equally sensitive radiological examination alternative for the CRC diagnosis for patients with and without alarm symptoms [111]. This method’s overall sensitivity is comparable to that of colonoscopy but is significantly lower for detecting polyps <8 mm [106].

Routine imaging-based diagnosis often limits the detection of cancer due to its small size or difficulty in separating it from soft tissues, which is particularly important for diagnosing metastases and assessing response to treatment [115]. A clinical challenge important for selecting and planning an appropriate management and treatment strategy is to perform a comprehensive clinical analysis that includes the use of the most recent imaging techniques combined with the assessment of tumor biomarkers and genetic features of the tumor [115]. The detection level of most conventional imagining techniques is insufficient to detect metastases. New techniques such as diffusion-weighted MRI (DW-MRI) or fibroblast activation protein inhibitor–positron emission tomography (FAPI-PET) is prospective due to high specificity and sensitivity, also in the case of extraperitoneal lesions [115,116,117,118].

An important marker that was supposed to help detect or predict the stadium of CRC is the carcinoembryonic antigen (CEA) concentration. A study in patients with abdominal symptoms, who have been ruled out after a complete colonoscopy, provides that CEA should not be considered to assist in the triage of patients with CRC [119]. The correlation between CEA levels and level of the tumor differentiation, diameter and staging is weak. In the majority of patients with and without colorectal cancer CEA levels may be within normal limits. Therefore, on this basis, it would not be ruled out the colorectal cancer diagnosis, and these patients should be investigated in detail [120]. Additionally, the preoperative serum level cannot indicate the specific stage and histopathological size of the CRC [121]. However, the CEA seems to be of substantial importance as a predictive and prognostic marker of relevance for choosing targeted therapy and for overall and progression-free survival in some types of CRC [122,123,124].

Once CRC is confirmed by histopathological examination, further diagnosis is determined individually depending on its findings. Additional diagnostics include imaging studies to assess the local stage, the presence of enlarged lymph nodes and distant metastases and the risk of obstruction. Additionally, based on the presence or absence of specific genetic biomarkers, individualized chemotherapy can be introduced, the efficacy of which may be higher compared to a standard procedure. In colon and rectal cancer appropriate for resection (non-metastatic), chest, abdominal, pelvic computed tomography (CT), pelvic magnetic resonance imagining (MRI), complete blood count, chemistry profile and CEA, enterostomal therapist as indicated for the preoperative marking of the site [125] and in rectal cancer proctoscopy endorectal ultrasound (if MRI is contraindicated or for superficial lesions) have to be considered [99]. In both colon and rectal cancer, the positron emission tomography-computed tomography (PET-CT) scan is not indicated, and in appropriate patients, fertility risk should be discussed [99,126]. In case of suspicion or proven metastatic synchronous adenocarcinoma (any T or N, and M1) the diagnosis should be extended by determination of tumor gene status for KRAS and B-RAF mutation and/or HER2 amplifications, testing microsatellite instability (MSI) and mismatch repair (MMR) and consider PET-CT scan (skull base to mid-thigh) and MRI od liver [99,125].

KRAS and BRAF encode a small G protein and a Ser/Thr protein kinase. They take part in regulating the mitogenic signaling cascade of the RAS/RAF/mitogenic-activated protein kinase (MAPK) or PI3K (phosphatidylinositol 3-kinase) pathways, which are activated by the epidermal growth factor receptor (EGFR). EGFR is responsible for stimulates critical processes involved in tumor growth and progression, including proliferation, angiogenesis, invasion and metastasis [127]. Mutations in the KRAS and B-RAF genes lead to hence constitutive activation of RAS/RAF proteins despite EGFR activation is blocked, and are considered to be an early event in CRC carcinogenesis with presence in about 20–50% cases [127,128]. A polymorphic tandem repeats of short nucleotide sequences distributed through the genome are microsatellites. These sequences are particularly prone to mutation due to polymerase errors, leading to frameshifts and base-pair substitutions during replication of DNA, resulting in shortening or extension of microsatellite regions in neoplastic cells. Mutation or silencing of MMR genes (such as MSH2, MSH6, PMS2 and MLH1) may cause MSI [129,130,131]. Patients with advanced CRC lacking KRAS or B-RAF mutations will prefer anti-EGFR therapy. In contrast, standard therapy based on 5-fluorouracil (5-FU) will be predisposed by testing for the presence or absence of chromosome deletions 18q and determining the tumor phenotype based on microsatellite instability (MSI) or microsatellite stability (MSS) [128,129,130].

In recent years, researchers have been working extensively to identify new biomarkers for the non-invasive diagnosis of CRC. Still, they currently may be considered as universal one about to predict the risk of invasion, metastasis occurrence or resistance to specific therapeutic regimens [132] and can be successfully translated into clinical practice [133]. There are a vast number of candidates for diagnostic biomarkers, depending on their types.

Abnormally methylated genes may affect the function of DNA repair (MGMT), apoptosis (BNIP3, DAPK and PCDH10), cell migration (vimentin and TIMP3), proliferation (CDKN2A, IGF2, MYOD1, RARβ2, SFRP1 and SFRP2), and differentiation (NDRG4), transcriptional regulation (GATA4 and TFAP2E) and others, and support prediction of clinical outcomes, such as treatment and survival prognosis, metastasis occurrence and therapy-resistant [134,135].

Protein biomarkers allow detecting CRC (MST1, serpin family, SEPT 9, leucine-rich alpha-2-glycoprotein 1, EGFR and inter-alpha-trypsin inhibitor heavy-chain family member 4) [133,136], staging existing cancer [137] and predict response for specific treatment (pEGFR for cetuximab response and PCBP1 and Cdk5 for oxaliplatin resistance) [133].

Detection of tumor circulating DNA (ctDNA) from dead cancer cells in body fluids may be applied to diagnosis, determine cancer type and grade, prognosis reoccurrence and treatment response [138]. Liquid biopsies like ctDNA are useful in detecting local tumors and distant metastatic, their types and non-invasive procedure [138,139]. However, these examinations are much adequate for high mutational burden tumors and associated with high false-positive/negative results [139]. The recent systematic review indicates that epigenetic ctDNA markers are potentially the most promising blood-based assay for CRC detection [140], and a high sensitivity and specificity screening test ctDNA SEPT9 methylation was approved to use. It is superior to CEA and FIT tests in asymptomatic population screening [141] and may be effectively used to exclude normal subjects [142].

MicroRNAs (miRNAs) are non-coding, endogenous, single-stranded RNA of 18–25 nucleotides length and can adversely regulate gene expression in the mechanism promoting the inhibition at the translation level or leading to the degradation of target mRNAs [143]. Differential expressions of various exosomal miRNAs, both alone and in panels, may constitute a potential biomarker for CRC diagnosis, making possible earlier diagnoses and a more personalized approach. Their role in clinical practice can be associated with diagnostic (miR-329, miR-181a, miR-199b, miR-382, miR-215 and miR-21), also in the early-stage (miR-125a-3p, miR-320c and miRNA-486-5p), prognostic (miR-181a-5p, miR-18a-5p and miR-18b-5p), tumor growth (miR-21, miR-23a, miR-92a and miR-1246) or predictive risk for high risk adenomas to transform in CRC (miR-21, miR-29a, miR-92a and miR-135b) and prognosis place of metastasis (miR-548c-5p and miR-328), predictive for adjuvant treatment and recurrence (miR-4772-3p) or stratification for chemotherapy (miR-21) [144]. Circulating miRNAs as novel biomarkers remain several challenges to be overcome before their clinical application. A further investigation regarding the origin and biological function of miRNAs is needed. Additionally, an explanation of the mechanisms through which miRNAs might be involved in the resistance to chemotherapy and other targeted therapies is required [145].

There has been an increased attempt to clarify the relationships between gut microbiota and colorectal cancer. Research results indicate that assessing different microbiota-related biomarkers may be a helpful non-invasive tool in CRC preventing, diagnosing and even treating. Some reviews and meta-analyses reported that specific species and gut bacterial dysbiosis might be related to CRC occurrence and observed in CRC patients and animal models [146]. As mentioned above, gut dysbiosis with high pathogenic microbiota metabolic activity may lead to deconjugation of bile acids and an increase in the level of secondary bile acids, e.g., deoxycholic acid, with an exert carcinogenic activity. Additionally, procarcinogenic and enterotoxin metabolites, such as sulfides, ammonia, phenols and nitrosamines, produced on the path of bacterial protein fermentation, amino acids degradation or reduction of dietary sulfate might be involved in CRC development [146]. In addition, gut microbes’ metabolic products may trigger inflammation response, produce reactive oxygen species, toxins or mediators (such as tumor necrosis factor alfa, interleukin-6 and cytokines), which may cause DNA damage and induced dysfunction or damage to epithelial cells [58]. High prevalence of *Fusobacterium nucleatum* [147], *Parvimonas micra* ATCC 33270, *Streptococcus anginosus*, *Parabacteroides distasonis* and other members of Proteobacteria were detected in samples of CRC and adenomas patients and present high discriminatory capacity in diagnosis [148]. In turn, bacterial as *F. nucleatum* [149,150,151] and *Bacteroides fragilis* were related with worse prognosis, while *Faecalibacterium prausnitzii* were common in the survival group [150]. A fascinating insight is that in *F. nucleatum* and *B. fragilis* high abundance group, KRAS and BRAF expression were more noticeable [150]. Recent work has suggested that fecal microbial composition and metabolites can module the response to chemotherapy or immunotherapy [152]. These opportunities might be related to stem cell transplantation’s effectiveness and modulating response to immunotherapy and treatment with immune checkpoint inhibitors. Microbial manipulation in the clinical setting by administrating targeted design probiotics may reduce proinflammatory and anti-inflammatory cytokines and locally alter immunity that positively impacts cancer therapies results [152]. Promising results are presented in the work of Poore et al. [153], in which using blood microbial DNA allows high cancer type discrimination between cancer and healthy patients. Assessment of microbial blood-based DNA in patients’ plasma, soon, may become a tool with a great potential for scheduling adequate treatment and expected therapeutic response [139]. However, there is insufficient evidence to recommend a microbiome-based test in place currently-used FIT or gFOBT as a non-invasive and inexpensive diagnostic tool in population-based screening programs [154]. Due to wide variability in bacterial species that may cause CRC, experts representing various fields need to collaborate to develop an inhibition strategy before progression to the neoplastic stage.

## 7. Conclusions

Further clinical studies are needed to understand the mechanisms of carcinogenesis, the impact of lifestyle, behavioral, environmental and genetic factors, or the synergistic action of the different aspects to increase preventive/treatment efficacy and patient survival with CRC.

Moreover, researchers are still searching for new tumor markers useful for diagnosis in primary and secondary care, but despite promising results, the evidence to date is insufficient. The ongoing research is critical to developing future strategies to control the burden of this disease through population-based prevention initiatives and demonstrates areas for further improvements in multidisciplinary cooperation.
